# Levels of Intestinal Inflammation and Fibrosis in Resection Specimens after Preoperative Anti-Tumor Necrosis Factor Alpha Treatment in Patients with Crohn's Disease: A Comparative Pilot Study

**DOI:** 10.1155/2020/6085678

**Published:** 2020-02-21

**Authors:** J. Torle, P. D. Dabir, U. Korsgaard, J. Christiansen, N. Qvist, A. El-Hussuna

**Affiliations:** ^1^Department of Surgery, Regional Hospital Randers, Midt-Jylland region, Randers, Denmark; ^2^Department of Pathology, Regional Hospital Randers, Midt-Jylland region, Randers, Denmark; ^3^IBD Care, Surgical Research Unit, Odense University Hospital, Southern University of Denmark, region Syd Danmark, Randers, Denmark; ^4^Department of Surgery, Aalborg University Hospital, Aalborg, Nord-Jylland region, Denmark

## Abstract

**Background:**

Strictures are a common complication in Crohn's disease (CD), found in more than 50% of patients. They are characterized by the excessive deposition of extracellular proteins in the tissue as a result of the chronic inflammatory process. The effect of anti-tumor necrosis factor alpha (TNF-*α*) therapy on the development of fibrosis is not yet fully understood.

**Aim:**

To investigate whether the degree of intestinal inflammation and fibrosis is correlated with preoperative anti-TNF-*α*) therapy on the development of fibrosis is not yet fully understood.

**Methods:**

This unblinded, prospective, single tertiary center, pilot cohort study included all adult patients with CD who underwent elective, laparoscopic, or open intestinal resection. Preoperative investigations included measurement of blood TNF-*α*) therapy on the development of fibrosis is not yet fully understood.

**Results:**

Histopathological specimens from 10 patients with CD who underwent ileocecal or ileocolic resections were retrieved. Four of those patients were on anti-TNF-*α*) therapy on the development of fibrosis is not yet fully understood. *α*) therapy on the development of fibrosis is not yet fully understood. *α*) therapy on the development of fibrosis is not yet fully understood. *p*=0.01). Anti-TNF-*α*) therapy on the development of fibrosis is not yet fully understood. *α*) therapy on the development of fibrosis is not yet fully understood. *α*) therapy on the development of fibrosis is not yet fully understood.

**Conclusions:**

Patients who underwent preoperative anti-TNF-*α* treatment had a higher fibrosis score than controls.*α*) therapy on the development of fibrosis is not yet fully understood.

## 1. Introduction

More than 50% of patients with Crohn's disease (CD) will develop intestinal fibrosis (stenosis and strictures) as a result of chronic inflammation [1].

Persistent and recurring tissue injury from inflammation, which is triggered and sustained by proinflammatory cytokines, leads to a continuous cycle of tissue breakdown and repair. This results in the accumulation of fibroblasts and myofibroblasts, which may lead to fibrosis as a result of extracellular matrix production [2].

Clinically, strictures may occur as a result of inflammation-mediated swelling of the intestinal wall, fibrotic occlusions, or a combination of both [3]. It is well known that immunosuppressive drugs, including TNF-*α* inhibitors, may reduce the occurrence of inflammatory strictures; however, their effect on the fibrotic reaction is not clear [4]. As chronic inflammation is closely associated with fibrosis and stricture formation, and considering the key role of TNF-*α* in initiating the inflammatory response [5], we hypothesized that anti-TNF-*α* therapy may lead to reduced fibrosis.

Intestinal macrophages have many important functions in maintaining gut homeostasis, but they also play a role in the development of inflammation. When identifying macrophages in tissue by immunohistochemistry, two widely used macrophage markers are CD68 and CD163 [6].

The aim of the present study was to investigate whether the degree of intestinal fibrosis, inflammation, and the number of macrophages are correlated with preoperative anti-TNF-*α* therapy in patients with CD undergoing bowel resection.

## 2. Materials and Methods

### 2.1. Study Design

This is an unblinded, prospective, noninterventional, pilot cohort study.

### 2.2. Study Hypothesis

Patients with CD who receive preoperative treatment with anti-TNF-*α* drugs will have less inflammation and a lower degree of intestinal fibrosis, assessed by CD163 concentration in the peripheral blood and histology, when compared to anti-TNF-*α* naïve patients.

### 2.3. Outcome Measures

The primary outcome measure was the degree of fibrosis. The secondary outcome was the grade of inflammation.

### 2.4. Inclusion Criteria

All adult patients with CD who were scheduled for elective small bowel or colon resection (open or laparoscopic) from a single tertiary center were included.

### 2.5. Exclusion Criteria

All patients with sepsis (abscess or fistula) or acute intestinal obstruction were excluded.

### 2.6. Extracted Data

This study is a continuation of the Inflammatory Bowel Disease Response study [7], in which resection specimens were obtained from patients undergoing elective ileocecal or ileocolic resections. Surgery and acquisition of the resection specimens were carried out as described previously [7].

Patient demographics, disease severity, nutritional status, comorbidities, preoperative medications, previous operations for CD, presence of preoperative intra-abdominal abscess or enteric fistula, preoperative nutritional support, disease duration, and disease localization were registered after obtaining oral and written informed consent. Blood samples were collected preoperatively and at 6, 24, and 48 hours after the surgical incision. Laboratory workup included serum analyses for TNF-*α*, interleukin- (IL-) 6, IL-8, IL-10, and IL-17A, in addition to white blood cell count, total iron-binding capacity, and C-reactive protein, albumin, hemoglobin, D-dimer, and cortisol levels. The drug concentration in the serum was measured in addition to the antidrug antibodies titer.

**Figure 1:**
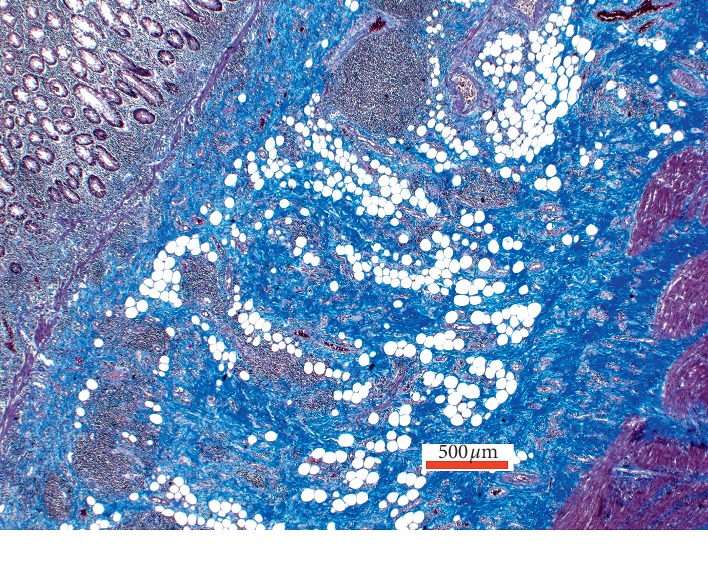
Example of fibrosis stained blue in Masson Trichrome special stain.

Out of the 46 patients included in the IBD Response study, bowel specimens from 10 patients with CD met the inclusion criteria and were eligible for analysis. Four of the 10 included patients had received TNF-*α* treatment preoperatively, with the last dose administered 1–9 weeks prior to surgical intervention. Representative hematoxylin and eosin (H&E)-stained slides from each of the resection specimens were chosen for further analysis. Fibrosis was assessed by Masson trichrome (MT) staining. Examples of fibrosis stained blue in Masson Trichrome special stain are shown in Figure 1.

To assess the number of macrophages, the immunohistochemical markers CD68 and CD163 were used. The slides were digitalized using a Hamamatsu slide scanner™ and image processing was subsequently performed in Visiopharm™. For both markers, deparaffinized sections were stained in a Ventana BenchMark ULTRA XT automatic stainer using 3,3′-diaminobenzidine (DAB) (OptiView universal DAB IHC detection kit; Ventana BioTek System, Tucson, AZ, USA), performed according to the manufacturer's instructions. Briefly, sections were demasked with cell conditioning buffer (CC1, Ventana Medical Systems, Tucson, AZ, USA) for 8 min followed by 16 min, and endogenous peroxidase activity was blocked with the UltraView inhibitor. Primary antibodies against CD68 (PREP KIT 54) and CD163 (CD163 [MRQ-26]) were applied for 32 min at 36°C. Sections were then incubated with OptiView horseradish peroxidase (HRP) multimer (Ventana BioTek Systems), containing a mixture of HRP-labeled goat anti-mouse and goat anti-rabbit antibodies. Bound antibodies were visualized by incubation with OptiView hydrogen peroxide substrate and DAB chromogen, and counterstained with hematoxylin. Positive signals for both CD68 and CD163 markers were evidenced by a brown color reaction in the cell cytoplasm.

Slides were evaluated by two consultant pathologists (PDD and JJC) and one trainee pathologist (UK) to minimize interobserver variation. The pathologists were blinded to the patients' clinical data and diagnosis. The three independent observers graded the degree of fibrosis on the MT-stained slides on a scale of 1–4. The grading was performed semi-quantitatively by assessing the amount of blue-stained fibers in the representative areas.

Acute and chronic inflammation was assessed in all four layers of the intestinal wall, and graded as absent, mild, moderate, or severe. The inflammation grades were determined semi-quantitatively by eyeballing, with a triple head microscope. Disagreements were resolved by consensus, and grades were assigned a numerical value from 0–3 for statistical analysis. The grades were defined as follows: absent (0), no traces of inflammation; mild (1), few inflammatory cells (mild acute inflammation in mucosa was defined as neutrophils in the lamina propria); moderate (2), moderate amount of inflammatory cells (moderate acute inflammation in mucosa was defined as the presence of cryptitis); and severe (3), many inflammatory cells (severe acute inflammation in mucosa was defined as the presence of crypt abscess).

Areas of ulceration were excluded from the analysis.

**Figure 2:**
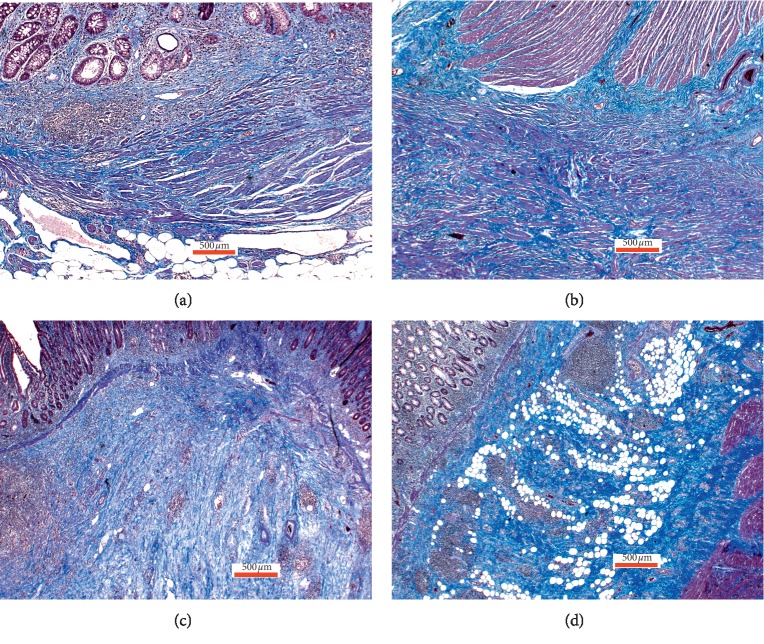
Example of cell cytoplasmatic staining of macrophages using the CD68 immunohistochemical marker.

The immunohistochemical slides were assessed independent to the HE-stained sections in a blinded manner. Positive immunohistochemical staining was defined as cytoplasmic staining for CD68 and CD163. To assess the number of CD68- and CD163-positive cells, the areas of mucosa, submucosa, and muscularis propria deemed to contain the most CD163-positive cells under 4× magnification were selected. The CD68 and CD163 slides were then aligned, and each observer counted the number of positive CD68 and CD163 cells in the selected area (110,000 *μ*m^2^) at 20x magnification. A representative image of CD68 staining is presented in Figure 2.

### 2.7. Statistical Analysis

For the fibrosis analysis, the mean value of each independent observers' scores were calculated and used as the final result. Student's *t*-test was applied for all observations. The kappa score for interobserver agreement was calculated using Fleiss' kappa for more than two raters. For the univariate analysis, Pearson's chi-square and Fisher's exact tests were employed. Continuous data were compared using the Mann–Whitney *U* test and one-way ANOVA. The relationship between continuous variables was analyzed using Pearson's chi-square correlation. Due to the small sample size of this pilot study, multivariate logistic regression was not used. SPSS version 19 software (IBM SPSS Statistics for Windows, Version 22.0; Armonk, NY: IBM Corp.2010) was used for all statistical analyses.

## 3. Results

### 3.1. Study Population Characteristics

Patients' characteristics are shown in Table 1. The mean age was 40.10 (±12.97 SD) years. Five patients were female. None of the patients had comorbidities.

### 3.2. Drug Levels at the Time of Operation

Four patients who underwent preoperative treatment with anti-TNF-*α* received their last dose 1–9 weeks prior to bowel resection. Three of the four patients who were on anti-TNF-*α* treatment had no detectable drug concentration at the time of operation. One patient was positive for antidrug antibodies at the time of operation, although this patient had an undetectable drug concentration.

### 3.3. Fibrosis

The patients' fibrosis scores are presented in Table 2. Patients on anti-TNF-*α* treatment had a higher fibrosis score compared to controls (*p*=0.01). There were no significant relationships between the number of weeks of administration of anti-TNF-*α* agents and the fibrosis score, CD68 or CD163 levels. The fibrosis score was not correlated with CD68 or CD163 levels. Preoperative and 6 h postoperative TNF-*α* concentrations were not correlated with the fibrosis score. The disease duration was not correlated with the degree of fibrosis.

### 3.4. Grade of Inflammation

Grades of acute and chronic inflammation are presented in Table 2. Patients on anti-TNF-*α* treatment showed no difference in inflammation compared to the controls. Neither the duration of anti-TNF-*α* treatment nor the anti-TNF-*α* drug concentration was associated with the grade of inflammation.

### 3.5. Macrophage Markers CD68 and CD163

The presence of CD68 and CD163 in the submucosa of patients with CD were significantly correlated (*p*=0.014), but not in the mucosa or muscularis propria. The concentrations of inflammatory markers (TNF-*α*, IL-6, IL-8, IL-10, IL-17, and cortisol) showed no significant correlations with CD68 or CD163, whether in the mucosa, submucosa or muscularis propria. Anti-TNF-*α* treatment was not associated with an increase in CD68 or CD163. No significant correlations were observed between preoperative C-reactive protein levels and fibrosis score, CD68 or CD163.

## 4. Discussion

There was no significant relationship between the duration of anti-TNF-*α* administration and the fibrosis score. This might be explained by differences in disease duration, the development of fibrosis prior to medication, or genotypic and phenotypic differences between patients. An allele that could lead to overexpression of the main profibrotic mediator, TGF-*β*, has been identified [8], which could lead to more rapid development of fibrosis and strictures. Another explanation could be that inflammation and fibrosis are initially linked in the pathogenesis, but later become two independent processes [9]. This might explain why fibrosis cannot be reversed by anti-inflammatory drugs.

The duration of anti-TNF-*α* treatment was not significantly correlated to either of the macrophage-associated receptors (CD68 and CD163). CD163 serves as a marker for activated macrophages in its soluble form [10]. Although the CD163 marker has been linked to the anti-inflammatory M2 macrophage phenotype, CD163-positive macrophages have been found to outnumber CD68-positive cells in a range of inflammatory events, including those that are associated with CD [6]. Dige et al. found that administration of anti-TNF-*α* led to a significant reduction in soluble CD163 when compared to biologic-naïve controls [10]. In the present study, we did not find such a correlation. This may be ascribed to the fact that plasma concentrations, rather than tissue concentrations, were measured in the Dige et al. study [10]. In addition, none of the patients had detectable drug levels at the time of operation, which might also explain why CD163 concentrations were not attenuated.

In order to measure the number of macrophages, two cellular markers, CD68 and CD163, were chosen. CD68 is a pan-macrophage marker [11], whereas CD163 is a macrophage-specific scavenger receptor with anti-inflammatory abilities in M2 macrophages. When macrophages are activated, CD163 proteins are shed into the bloodstream [10]. By measuring soluble CD163, one can monitor the number of activated macrophages in the host. Consequently, elevated levels of CD163 are associated with conditions with a heightened inflammatory response, such as CD [10]. Using this method, however, no correlation between fibrosis and the number of macrophages was found. Additionally, no correlations were found between various cytokines (TNF-*α*, IL6 IL8, IL10, and IL17) and cortisol and CD68 and CD163. If this holds true in a larger population, it could indicate that macrophages play a more active role in inflammation than in the development of fibrosis. Alternatively, the question must be raised as to the accuracy of these markers for measuring the degree of fibrosis.

The lack of association between treatment with the anti-TNF-*α* drug and a decrease in inflammation is remarkable, as its main effect is to inhibit the proinflammatory cytokine TNF-*α*. This could indicate that the effect of the drug is subeffective or ineffective in the target tissue. Previous studies have concluded that inflammatory processes may be confined to the fibrostenotic tissue, which may be beyond the reach of anti-TNF-*α* drugs [12]. Also, of the patients in the treatment group, half had received their last dose 9 weeks prior to surgery, which might be too long to detect a decrease in inflammation. If this is the case, it may explain why we observed no reductions in fibrosis or in macrophage activity.

The increase in fibrosis found in this study may seem counterintuitive considering its effectiveness against inflammation. This has been reported in other studies, and the general explanation is that TNF-*α* counterbalances the effects of TGF-*β*. Inhibition of TNF-*α* might therefore promote the profibrotic signaling cascade [13]. Although previous studies have found a significant decrease in inflammation in the treatment group, there was a significant increase in pauci-inflammatory fibrosis in the muscularis mucosae, as well as a trend toward increased fibrosis in the muscularis propria, albeit not statistically significant. This was ascribed to the classic “lattice-like” healing pattern also seen in endoscopy after anti-TNF-*α* treatment [13]. Since its introduction, theories related to the role of anti-TNF-*α* in the development of fibrosis have been subject to debate. Although several studies conducted on large populations have failed to produce evidence that support such a claim, concerns regarding the drug's potential in causing fibrosis and strictures in these patients still remain [13].

We suspect that the increased fibrosis score observed in patients with CD on anti-TNF-*α* treatment in the current study indicates a nonresponse to the treatment because a response would have limited inflammation and fibrosis. This hypothesis is to be investigated in a large prospective cohort study.

## 5. Limitations

This study was a pilot study that can serve as a template for future larger-scale studies. The two groups were not comparable with regard to age, gender, disease duration, preoperative events, and surgical approach. The patients in the control group seemed to be more sick (older, longer disease duration, and open surgery), which would translate to more inflammation and fibrosis. The study population was a group of patients in whom the medical treatment was not sufficient to avoid operation, and nonresponders to anti-TNF-*α* treatment may differ to responders with regard to the formation of fibrosis. Logistic regression analysis to adjust for confounders was considered, but due to the small sample size, and after consulting a biomedical statistician, this was deemed inappropriate.

## 6. Conclusion

Patients who received preoperative anti-TNF-*α* treatment had a higher fibrosis score compared to controls. A significant correlation between the duration of drug administration and fibrosis score could not be found. Furthermore, there was no correlation between the duration of drug administration and the number of CD68 and CD163 macrophage receptors. The degree of acute and chronic inflammation was not significantly different between the two groups. There is a need for a large prospective study.

## Figures and Tables

**Table 1:** Preoperative and intraoperative characteristics of 10 patients with Crohn's disease treated with anti-TNF-*α* compared to anti-TNF-*α* naïve patients.

Patients' characteristics	Anti-TNF-*α* treatment 4/10 patients (40%)	Anti-TNF-*α* naïve 6/10 patients (60%)	Univariate analysis
Age (years; mean ± SD)	27.4 ± 10.25	48.5 ± 5.28	*p*=0.003
Female	3/4 (75%)	2/6 (33.3%)	Ns
Body mass index (kg/m^2^; mean ± SD)	26.58 ± 11.8	22.5 ± 2.17	Ns
Smoking, *n* (%):			Ns
Non- or ex-smoker	3/4 (75%)	6/6 (100%)	
Smoker	1/4 (25%)	0/6 (0%)	
Steroids, *n* (%)	0	3/6 (50%)	Ns
Immunomodulators, *n* (%)	1/4 (25%)	2/6 (33.3%)	Ns
Disease localization (Montreal classification)			Ns
L2	1/4 (25%)		
L3	3/4 (75%)		
Harvey–Bradshaw Index > median (7.5), *n* (%)	1/4 (25%)	3/6 (50%)	Ns
Disease duration (years; mean ± SD)	5 ± 3.16	14.33 ± 9.05	Ns
Disease phenotype (Montreal classification)			Ns
B2 (stricturing)	4/4	3/6 (50%)	
B3 (penetrating)	0	3/6 (50%)	
Previous laparotomy/laparoscopy for CD	1/4 (25%)	3/6 (50%)	Ns
Preoperative parenteral nutrition, *n* (%)	0	1/6 (16.7%)	Ns
Preoperative sepsis	0	2/6 (33.3%)	Ns
Preoperative CRP mg/L (mean ± SD)	32.18 ± 37.5	19.35 ± 19.28	Ns
Preoperative TNF-*α* ng/L (mean ± SD)	1.22 ± 2.14	0.37 ± 0.35	Ns
Access to abdomen, *n* (%)			*p*=0.005
Laparoscopic	4/4	0	
Open	0	6/6	
Type or resection, *n* (%)			Ns
Small bowel resection	0	2/6 (33.3%)	
Ileocolic resection	4/4^*∗*^	4/6 (66.7%)^*∗*^	

All operations were performed with a specialist surgeon in charge. Anti-TNF-*α*, anti-tumor necrosis factor alpha drugs; Ns, nonsignificant; CRP, C-reactive protein; SD, standard deviation. ^*∗*^In each group that underwent ileocolic resection, one patient also received colectomy.

**Table 2:** Drug administration, fibrosis score, and inflammation grade of patients with Crohn's disease.

Birth year	Gender (m/*f*)	Anti-TNF-*α* dose (mg)	TPS (weeks)	Fibrosis score			Acute inflammation grade				Chronic inflammation grade			
				*P*1	*P*2	*P*3	Mu	SM	MuP	SS	Mu	SM	MuP	SS
1997	f	40	1	4	3	4	2	1	0	0	3	3	1	2
1993	m	100	9	3	3	3	1	0	1	0	1	2	1	1
1974	m	0	—	2	2	3	2	1	0	0	2	2	2	1
1963	m	0	—	1	1	1	3	0	1	0	1	3	2	1
				1	1	2	1	0	0	0	1	1	1	0
1965	f	0	—	2	3	2	2	0	0	1	1	2	0	1
1962	m	0	—	2	3	4	1	0	0	1	2	3	2	2
				2	1	2	3	0	1	0	2	3	1	2
1982	f	40	1	3	3	4	1	0	0	1	2	2	1	1
1962	f	0	—	2	3	3	3	1	1	0	2	3	2	3
1972	m	0	—	1	2	1	3	1	0	1	1	1	0	1
1975	f	100	9	4	4	4	2	0	1		2	0	1	
				3	3	4	1	0	0	0	0	0	0	1

TPS, time prior to surgery; P1–3, pathologists 1–3; Mu, mucosa; SM, submucosa; MuP, muscularis propria; SS, subserosa.

## Data Availability

The data used to support the findings of this study are available from the corresponding author upon request.

## Additional Points

*Perspectives.* Histopathological examination of fibrosis and inflammation might indicate whether the patient will respond to biological treatment.
